# Intrinsic Tumor Aggressiveness Dictates Hypoxia-Driven Metabolic Programs in Hepatocellular Carcinoma

**DOI:** 10.3390/ijms27073069

**Published:** 2026-03-27

**Authors:** Fabiola Milosa, Nicolò Giglioli, Rosina Maria Critelli, Francesco Dituri, Grazia Serino, Serena Mancarella, Erica Villa

**Affiliations:** 1 EpatoGastro Lab-RBA Labs, Department of Medical, Dental and Morphological Sciences with a Focus on Transplantation, Oncology and Medicine, University of Modena and Reggio Emilia, Via del Pozzo 71, 41125 Modena, Italy; fabiola.milosa@unimore.it (F.M.); nico.giglio@unimore.it (N.G.); rosinamaria.critelli@unimore.it (R.M.C.); 2 National Institute of Gastroenterology IRCCS “Saverio de Bellis”, Research Hospital, Via Turi 27, 70013 Castellana Grotte, Italy; francesco.dituri@irccsdebellis.it (F.D.); grazia.serino@irccsdebellis.it (G.S.); serena.mancarella@irccsdebellis.it (S.M.); 3 Department of Medical, Dental and Morphological Sciences with a Focus on Transplantation, Oncology and Medicine, University of Modena and Reggio Emilia, Via del Pozzo 71, 41125 Modena, Italy

**Keywords:** hypoxia, tumor aggressiveness, hepatocellular carcinoma, metabolic reprogramming, glycolysis, patient-derived spheroids

## Abstract

Hypoxia, a hallmark of hepatocellular carcinoma (HCC), regulates metabolic reprogramming, tumor progression, and therapy resistance. Although hypoxia-induced glycolytic changes are recognized, it remains unclear how intrinsic tumor aggressiveness influences the magnitude and plasticity of metabolic and transcriptional responses to oxygen deprivation. In this study, we investigated the effects of chronic hypoxia (1% O_2_ for 48 h) in spheroids generated from two immortalized (HepG2, Hep3B) and two patient-derived HCC cell lines with distinct aggressiveness (HLC19, HLC21). The metabolic activity, energetic status, proliferation, and expression of hypoxia- and metabolism-related genes were assessed, with oxygen levels monitored to validate experimental conditions. It has resulted that immortalized HCC spheroids displayed similar metabolic and transcriptional responses to hypoxia, with enhanced glycolytic activity but limited phenotypic plasticity. Primary HCC spheroids exhibited aggressiveness-dependent differences. Aggressive HLC19 cells showed a pre-established glycolytic phenotype, stable ATP levels, sustained proliferation, and minimal transcriptional remodeling under hypoxia. Less aggressive HLC21 cells relied on the delayed glycolytic activation and induction of hypoxia-responsive genes to maintain viability. Clustering analyses indicated that metabolic strategies, rather than absolute activity, aligned with tumor aggressiveness. These findings suggest that intrinsic tumor aggressiveness shapes hypoxia-driven metabolic programs in HCC and supports the relevance of patient-derived 3D models for studying metabolic adaptation.

## 1. Introduction

Hepatocellular carcinoma (HCC) is the most common primary liver tumor, accounting for approximately 70–90% of all liver cancers [1]. Owing to its high incidence, mortality, and marked biological heterogeneity, HCC represents a valuable model for investigating the molecular mechanisms driving tumor development and progression. A distinct feature of solid tumors, including HCC, is “hypoxia”, characterized by regions of insufficient oxygen supply within the tumor microenvironment [2,3]. In a healthy liver, the extensive sinusoidal network ensures adequate oxygenation, whereas in HCC the combination of aberrant vascularization and elevated metabolic demand creates a chronically hypoxic milieu [4]. Hypoxia is a major driver of tumor aggressiveness, biological heterogeneity and treatment resistance [5,6,7]. Clinically, hypoxia following transarterial chemoembolization promotes the selection of more aggressive tumor clones and fosters vascular remodeling, contributing to disease progression and recurrence [8,9].

At the cellular level, hypoxia activates multiple adaptive programs, particularly angiogenic and metabolic pathways [10]. Central to this response is hypoxia-inducible factor-1α (HIF-1α), which drives metabolic reprogramming toward glycolysis by upregulating glucose transporters and glycolytic enzymes [11]. This shift—known as the “Warburg effect”—increases glucose uptake and lactate production even in the presence of oxygen, promoting the acidification of the extracellular microenvironment and enhancing tumor invasiveness, as well as therapy resistance and immune evasion [7,12,13]. Given the central role of hypoxia in tumor progression, the accurate in vitro modeling of this microenvironment is crucial. Three-dimensional (3D) cell cultures such as spheroids recapitulate the key architectural features of solid tumors, including oxygen and nutrient gradients that resemble those observed in vivo [14,15,16,17]. Here, we investigated whether intrinsic tumor aggressiveness influences hypoxia-driven metabolic reprogramming in HCC. We compared four spheroid models cultured under chronic hypoxia (1% O_2_ for 48 h): two immortalized HCC lines with different aggressive phenotypes (HepG2 and Hep3B) [18,19,20] and two patient-derived primary HCC cell populations (HLC19 and HLC21) exhibiting distinct in vivo behavior. This phenotype-driven design enables controlled mechanistic comparisons while minimizing confounding variability between models. We hypothesized that intrinsic tumor aggressiveness determines whether hypoxia induces adaptive reprogramming or reveals a pre-adapted metabolic state.

## 2. Result

### 2.1. Validation of Hypoxic Conditions: Rapid Oxygen Decline and Long-Term Stability in Cell Cultures

Oxygen concentration in the culture medium was monitored at baseline and after 2, 4, 24 and 48 h under both normoxic and hypoxic conditions. At baseline, oxygen levels were similar across all conditions (approximately 21.75%).

**Figure 1:**
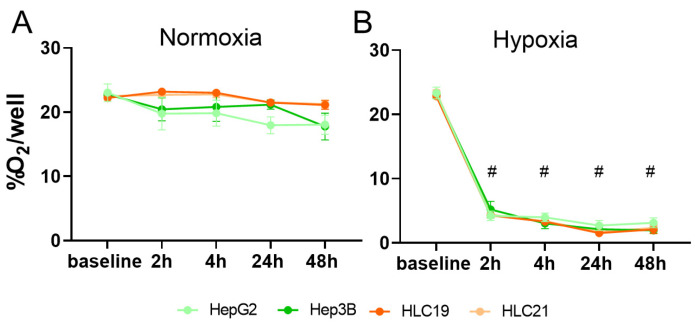
**Oxygen levels under normoxic and hypoxic conditions.** Percentage of oxygen per well under normoxic (21% O_2_) (**A**) and hypoxic (1% O_2_) (**B**) conditions in HepG2, Hep3B, HLC19, and HLC21 cultures, measured at baseline and after 2, 4, 24, and 48 h. Under normoxia, oxygen levels remained stable across all time points without statistically significant differences, whereas under hypoxia they rapidly declined within first 2 h and subsequently stabilized at low levels up to 48 h. Statistical significance: # *p* < 0.0001, 2 h, 4 h, 24 h and 48 h versus baseline for each group.

Under normoxia, oxygen concentrations remained stable (19–21%) throughout the experiment with no significant differences among cell lines (Figure 1A). Under hypoxia, oxygen levels dropped rapidly within the first 2 h, reaching approximately 4.0% in all cell lines, both immortalized and primary, and then stabilized between 1 and 2% up to 48 h (Figure 1B). These results confirm the effectiveness of the experimental setup in rapidly inducing and stably maintaining hypoxic conditions over time.

### 2.2. Differential Glucose Uptake and Lactate Secretion in HCC Cell Lines Reveal Metabolic Plasticity Linked to Tumor Phenotype

To investigate glucose metabolism in both immortalized and primary HCC cell lines, extracellular glucose and lactate levels were measured in the culture medium at baseline (cell-free medium) and after 2, 4, 6, 24, and 48 h of culturing. Glucose uptake and lactate secretion were calculated as the difference between metabolite concentrations in fresh medium and those measured in the culture supernatants at each time point.

#### 2.2.1. HepG2 Exhibits Greater Metabolic Plasticity Compared to Hep3B Cells

**Figure 2:**
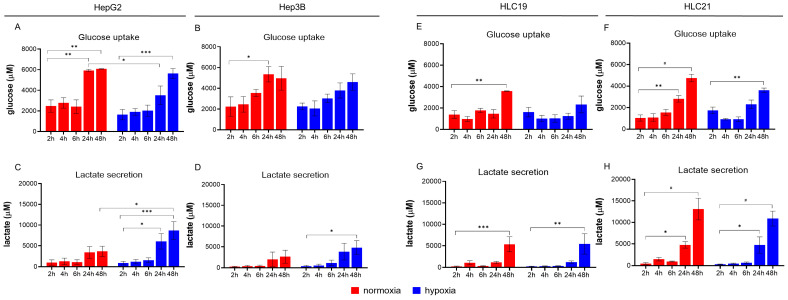
**The metabolic assessment of glucose uptake and lactate production in all spheroids.** (**A**,**B**,**E**,**F**) Glucose uptake, calculated as the difference between extracellular glucose in cell-containing samples and cell-free medium, measured at 2, 4, 6, 24 and 48 h under normoxic or hypoxic conditions ((**A**): HepG2; (**B**): Hep3B; (**E**): HLC19; (**F**): HLC21). (**C**,**D**,**G**,**H**) Lactate secretion, quantified from culture supernatants at the same time points as an indicator of glycolytic activity ((**C**): HepG2; (**D**): Hep3B; (**G**): HLC19; (**H**): HLC21). Glucose and lactate levels are expressed in μM. Together, these measurements capture the temporal and oxygen-dependent metabolic responses of the two immortalized and two primary HCC lines. Statistical significance: * *p* < 0.05, ** *p* < 0.01; *** *p* < 0.001, # *p* < 0.0001.

Under normoxic conditions, HepG2 spheroids showed a significant increase in glucose uptake after 24 and 48 h compared to 2 h, whereas under hypoxia, a significant increase was detected only at 48 h (Figure 2A). Notably, glucose uptake at 24 h was significantly higher in normoxia than in hypoxia. In contrast, Hep3B spheroids exhibited only a non-significant upward trend, with a modest peak at 24 h under normoxia and no significant changes in either oxygen condition (Figure 2B).

Lactate production in HepG2 spheroids increased over time, paralleling the glucose uptake pattern. This effect was more pronounced under hypoxia, with significantly higher lactate levels at 48 h compared to normoxia (Figure 2C). Hep3B cells showed a similar temporal trend, although overall lactate production was lower (Figure 2D). In these cells, no significant differences between normoxic and hypoxic conditions were observed, except for a modest increase at 48 h compared to 2 h under hypoxia. Collectively, these data indicate that both immortalized lines undergo time-dependent metabolic adaptation, with HepG2 displaying greater metabolic plasticity than Hep3B.

#### 2.2.2. More Aggressive HLC19 Cells Show Earlier and Stronger Glycolytic Activation than Less Aggressive HLC21 Cells

Primary HCC cells exhibited overlapping yet distinct metabolic trends. The more aggressive HLC19 line showed a significant increase in glucose uptake at 24 h and 48 h under normoxic conditions, similar to HepG2 but at lower absolute levels. Under hypoxia, glucose uptake increased significantly only at 48 h (Figure 2E). Lactate production in HLC19 followed a comparable trend, with a progressive increase over time under both oxygen conditions, consistent with active glycolysis and enhanced metabolic flexibility (Figure 2G). In contrast, HLC21 cells showed no significant change in glucose uptake until 48 h under normoxia (Figure 2F), and lactate levels increased only at 48 h, irrespective of oxygen availability (Figure 2H). Overall, these findings indicate that primary HCC cells, while sharing some metabolic features with immortalized lines, exhibit distinct temporal and oxygen-dependent glucose and lactate dynamics. Notably, HLC19 cells show earlier and more pronounced glycolytic activation, whereas HLC21 cells show a delayed and attenuated response, consistent with a less metabolically active phenotype.

#### 2.2.3. Metabolic Correlations Reveal Cellular Programs Linked to Tumor Aggressiveness

To further examine the coupling between glucose consumption and lactate secretion, we performed Pearson correlation analyses in all four HCC cell lines under normoxic and hypoxic conditions (Table 1). A strong positive correlation emerged, exclusively in primary cells, under both hypoxia (HLC19 r = 0.789, *p* < 0.001; HLC21 r = 0.933, *p* < 0.001) and normoxia (HLC19 r = 0.884, *p* < 0.001; HLC21 r = 0.662, *p* = 0.007). Among immortalized lines, a significant positive correlation was detected only in HepG2 under hypoxia (r = 0.717, *p* < 0.001).

**Figure 3:**
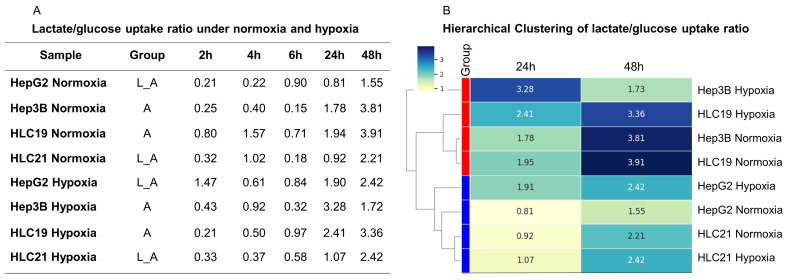
**The lactate/glucose uptake ratio in HCC cell lines with different aggressiveness under normoxic and hypoxic condition.** (**A**) The ratio was calculated at 2, 4, 6, 24 and 48 h for aggressive (Group A) (Hep3B, HLC19) and less aggressive (Group L_A) (HepG2, HLC21) phenotypes. Ratios exceed a value of 2 (which means that all glucose is converted into lactate) from 24 h onward particularly in aggressive cells, indicating sustained glycolytic activity, highlighting metabolic differences among cell lines and the influence of oxygen availability. (**B**) The hierarchical clustering of lactate-to-glucose uptake ratios in HCC cell lines (red group for aggressive cells, blue group for less aggressive ones). The lactate-to-glucose uptake ratio was calculated for HepG2, Hep3B, HLC19, and HLC21 at 24 h and 48 h under normoxic and hypoxic conditions. Ratios consistently exceeded 2 from 24 h onward, indicating enhanced glycolytic activity in all cell lines. The hierarchical clustering of these ratios revealed grouping based on the intrinsic tumor phenotype, with Hep3B clustering with HLC19 and HepG2 clustering with HLC21, suggesting shared metabolic programs associated with specific aggressiveness profiles.

To assess the efficiency of lactate production relative to glucose consumption, the lactate-to-glucose uptake ratio was calculated (Figure 3A). This ratio revealed clear metabolic differences among HCC cell lines according to aggressiveness and oxygen availability. Under normoxia, aggressive cell lines (Hep3B and HLC19) exhibited markedly higher ratios than less aggressive counterparts at both 24 h and 48 h (Hep3B: 1.78 and 3.81; HLC19 1.94 and 3.91), indicating a stronger glycolytic phenotype. In contrast, HepG2 and HLC21 showed lower ratios at the same time points (24 h: 0.81 and 0.92; 48 h: 1.55 and 2.21, respectively), suggesting a less pronounced glycolytic shift. Under hypoxia, this aggressiveness-dependent pattern was largely maintained. HLC19 sustained a high ratio at both 24 h (2.41) and 48 h (3.36), whereas Hep3B showed a transient peak at 24 h followed by a reduction at 48 h, suggesting early metabolic adaptation or feedback regulation. Less aggressive cells exhibited a progressive increase in their ratios under hypoxia, reaching the same values at 48 h (2.42 for each line). Overall, aggressive HCC cells (Hep3B and HLC19) displayed higher lactate-to-glucose ratios, particularly under hypoxic stress, supporting enhanced glycolysis as a hallmark of aggressive phenotypes. A clustering analysis of the ratios at 24 and 48 h revealed grouping based on the intrinsic tumor phenotype: Hep3B clustered with HLC19, while HepG2 clustered with HLC21, under both normoxic and hypoxic conditions (Figure 3B). These findings suggest the presence of shared metabolic pathways associated with specific aggressiveness profiles.

### 2.3. ATP Production Reveals Distinct Viability and Hypoxia Adaptation Profiles in Immortalized and Primary HCC Cells

**Figure 4:**
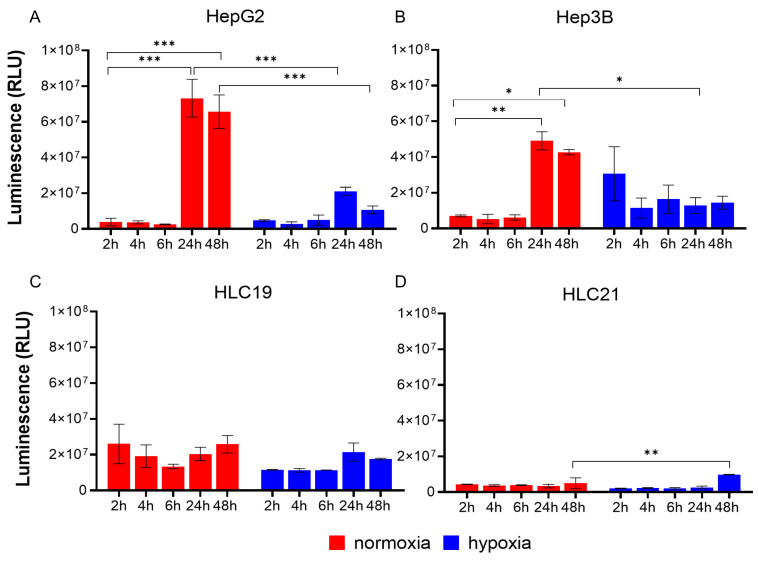
**ATP-based viability and metabolic activation of immortalized and primary HCC spheroids.** ATP-dependent luminescence (RLU) was measured in cellular lysates collected during metabolic assays as indicator of cell viability and energetic status. (**A**,**B**) Immortalized HCC spheroids showing higher ATP levels under normoxic than hypoxic conditions ((**A**), HepG2; (**B**), Hep3B). (**C**,**D**) Primary HCC spheroids displaying relatively stable ATP levels over time ((**C**), HLC19; (**D**), HLC21). Statistical significance: * *p* < 0.05, ** *p* < 0.01; *** *p* < 0.001.

ATP production was measured in spheroid lysates collected during the metabolic assays as an indicator of cellular viability and energetic status. Among immortalized cell lines, HepG2 spheroids cultured under normoxic conditions showed a significant increase in ATP-dependent luminescence at 24 and 48 h compared with 2 h, with ATP levels higher under normoxia than under hypoxia (Figure 4A). In Hep3B spheroids, ATP levels were comparable to those observed in HepG2 under both hypoxic and normoxic conditions, with no marked differences over time (Figure 4B).

In primary HCC cells, the more aggressive HLC19 exhibited higher ATP levels than immortalized cells at early time points, which remained relatively stable over time (Figure 4C). In contrast, HLC21 spheroids displayed lower overall ATP levels, with a significant increase under hypoxia compared with normoxia at 48 h, suggesting the late activation of adaptive response to hypoxia (Figure 4D).

### 2.4. Cells Show Divergent Ki67 Expression and Proliferative Dynamics Under Hypoxia

To assess proliferative activity under hypoxia and to also visualize the spatial distribution of proliferative cells, Ki67 expression was analyzed by immunofluorescence. In addition, the percentage of Ki67-positive cells was quantified to estimate spheroid capacity.

**Figure 5:**
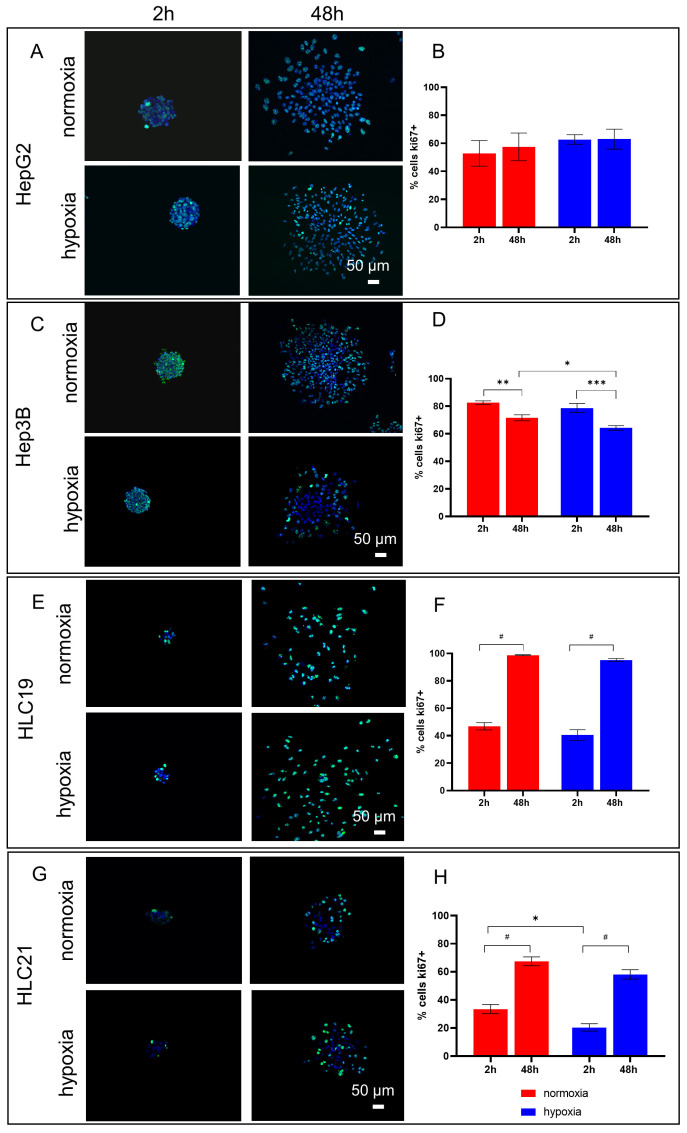
**Analysis of proliferative activity in HCC spheroids by Ki67 immunofluorescence.** Representative images at 2 and 48 h under normoxia and hypoxia of Ki67 staining (green) in HepG2 (**A**), Hep3B (**C**), HLC19 (**E**) and HLC21 (**G**) spheroids. Quantification of Ki67-positive cells in HepG2 (**B**), Hep3B (**D**), HLC19 (**F**) and HLC21 (**H**) spheroids in same conditions. (**A**,**B**) HepG2 spheroids: no significant differences in Ki67 expression, with approximately 60% of cells being Ki67-positive at both time points. (**C**,**D**) Hep3B spheroids: strong Ki67 staining at 2 h in both conditions and reduction at 48 h, with higher proportion of Ki67-positive cells under normoxia compared with hypoxia. (**E**,**F**) HLC19 spheroids: Ki67-positive cells distributed throughout spheroid, with significant increase at 48 h (approximately 90% of cells) under both conditions. (**G**,**H**) HLC21 spheroids: Ki67 expression predominantly localized at spheroid periphery, with approximately 70% of cells being positive after 48 h under both conditions. Nuclei are stained with DAPI (blue). Statistical significance: * *p* < 0.05, ** *p* < 0.01; *** *p* < 0.001, # *p* < 0.0001.

In HepG2 spheroids, no differences in Ki67 expression were observed between normoxia and hypoxia after 2 h, and this pattern was maintained at 48 h, with approximately 60% of cells remaining Ki67-positive (Figure 5A,B). Hep3B spheroids exhibited strong Ki67 staining after 2 h in both oxygen conditions. At 48 h, Ki67 expression decreased, with a higher proportion of Ki67-positive cells at 48 h under normoxic conditions compared with hypoxia (Figure 5C,D).

In primary HCC spheroids, both cell lines showed a significant increase in the percentage of Ki67-positive cells at 48 h under normoxic and hypoxic conditions, reaching approximately 90% in HLC19 and 70% in HLC21 (Figure 5F,H). An analysis of Ki67 spatial distribution revealed distinct patterns between the two primary models: in HLC21, Ki67 expression was predominantly localized to peripheral cells, whereas in HLC19 spheroids, Ki67-positive cells were distributed throughout the entire spheroid (Figure 5E,G).

### 2.5. HLC19 Cells Exhibit Enhanced Hypoxic Adaptation Compared to Less Aggressive HLC21 Cells

To facilitate phenotype-oriented interpretation, we directly compared more and less aggressive cell lines under hypoxia (Supplementary Figure S4) and normoxia (Supplementary Figure S5). While glucose uptake did not differ substantially between groups, lactate secretion was significantly higher in HLC19 than in HLC21 at 48 h. Moreover, aggressive HLC19 spheroids maintained higher ATP levels and proliferative activity. These findings reinforce the concept that aggressive primary HCC cells exhibit intrinsic metabolic pre-adaptation to hypoxic stress.

### 2.6. Differential Gene Expression Under Hypoxia in Immortalized and Primary Hepatic Cell Lines

**Figure 6:**
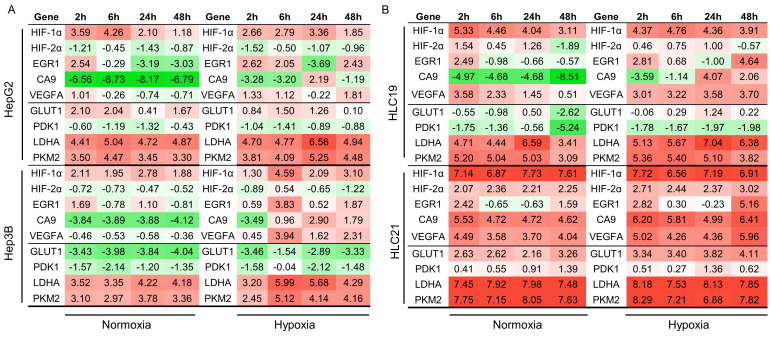
**A gene expression heatmap of immortalized (A) and patient-derived (B) cell lines under normoxic and hypoxic conditions.** A heatmap depicting the expression profiles of hypoxia-related (HIF-1α, HIF-2α, EGR1, VEGFA, CA9) and metabolism-associated genes (GLUT1, LDHA, PKM2, PDK1) in HepG2 and Hep3B cells (**A**) and HLC19 and HLC21 (**B**) exposed to normoxic or hypoxic conditions across the experimental time course. Gene expression was reported as the ratio of the target gene copy number to the reference gene (YWHAZ for HepG2 and Hep3B; POLR2A for HLC19 and HLC21), log_2_-transformed. The color scale ranges from red (strong upregulation) to green (strong downregulation), with intermediate intensities representing proportional expression changes.

Gene expression was analyzed by dPCR in all cell lines cultured under normoxic and hypoxic conditions for 2, 6, 24 and 48 h. Stability analysis confirmed YWHAZ and POLR2A as the most stable reference genes in immortalized and primary cells, respectively (Supplementary Figure S3). The absolute transcript levels (number of copies) of each target gene were normalized accordingly and subsequently log_2_-transformed. Hypoxia-related genes (HIF-1α, HIF-2α, CA9, VEGFA and EGR1) and metabolism-related genes (GLUT1, LDHA, PDK1 and PKM2) were evaluated. Heatmaps summarizing gene expression patterns under normoxia and hypoxia are shown for immortalized cell lines (HepG2 vs. Hep3B; Figure 6A) and primary cell lines (HLC19 vs. HLC21; Figure 6B).

#### 2.6.1. Immortalized Cell Lines

An analysis of gene expression heatmaps revealed no substantial differences between HepG2 and Hep3B under normoxic and hypoxic conditions (Figure 6A). HIF-1α was consistently upregulated in both cell lines at all time points, whereas HIF-2α remained downregulated even under hypoxia. Among HIF target genes, EGR1, VEGFA, and CA9 were induced exclusively under hypoxic conditions in both cell lines, albeit with distinct temporal dynamics: EGR1 showed early induction, whereas CA9 increased predominantly at later time points. VEGFA was stably upregulated over time.

Among metabolism-related genes, GLUT1 displayed the most pronounced divergence between HepG2 and Hep3B. GLUT1 was consistently upregulated in HepG2 across all time points, whereas it remained persistently downregulated in Hep3B under both oxygen conditions. In contrast, LDHA and PKM2 were steadily upregulated in both immortalized lines, showing stable expression over time, while PDK1 remained uniformly downregulated. Overall, immortalized cell lines did not exhibit coherent or phenotype-specific transcriptional response to hypoxia. Their gene expression profiles remained largely stable and similar across oxygen conditions, indicating limited transcriptional plasticity in response to environmental stress.

#### 2.6.2. Primary Cell Lines

In contrast, primary cell lines displayed marked transcriptional divergence (Figure 6B). The most prominent finding was the broad upregulation of both hypoxia- and metabolism-related genes in HLC21 compared with HLC19.

Notably, CA9 showed a distinct temporal and condition-dependent pattern: in HLC21, CA9 remained upregulated throughout the entire time course under both normoxia and hypoxia, whereas in HLC19 its induction was delayed and observed only at 24 and 48 h and exclusively under hypoxic conditions. Differences between primary cell lines were particularly pronounced for GLUT1 and PDK1, which were consistently downregulated in HLC19 but upregulated in HLC21 under both oxygen conditions. In contrast, LDHA and PKM2 were upregulated in both primary cell lines and remained stable over time. Collectively, these data demonstrate that primary HCC cells exhibit distinct and condition-dependent transcriptional responses to hypoxia that reflect differences in tumor aggressiveness.

## 3. Discussion

Our study investigated how chronic hypoxia [21] modulates metabolic, energetic, proliferative, and transcriptional programs in HCC models with different intrinsic aggressiveness. While hypoxia-driven metabolic reprogramming is a well-established feature of cancer [12,22,23], our aim was to determine whether the efficiency and dynamics of this adaptation are shaped by tumor malignancy, particularly in physiologically relevant in vitro systems [24,25,26].

HCC represents a paradigmatic model of hypoxia-driven adaptation, due to its aberrant vascularization and high metabolic demand, which expose tumor cells to fluctuating and often chronic low-oxygen conditions [27,28]. Hypoxia is closely linked to tumor progression, heterogeneity and poor prognosis. Clinically relevant interventions, such as transarterial chemoembolization, further exacerbate hypoxic stress, promoting clonal selection and vascular remodeling [9,24,25,26]. Despite extensive evidence linking hypoxia and aggressiveness in cancer [24,29], direct comparisons across HCC models of different malignancy—particularly including patient-derived cells—remain limited. By integrating immortalized and primary HCC spheroids [18,19,30], our study addresses this gap by providing a controlled comparative framework to explore how intrinsic tumor aggressiveness influences metabolic adaptation to hypoxia.

To model hypoxia, we exposed 3D spheroids to uniform low-oxygen conditions (1% O_2_) for 48 h [31]. Given the relatively small size of our spheroids (38 to 120 µm at baseline; 120 to 175 µm after 48 h), and the variable development of necrotic or hypoxic cores among the four cell lines over time, uniform hypoxic exposure ensured consistent oxygen deprivation across all cell populations [20,32]. The continuous monitoring of oxygen levels confirmed the stability of both normoxic and hypoxic conditions throughout the experiments. The activation of hypoxic signaling pathways was validated by a transcriptional analysis of canonical hypoxia markers. In immortalized cell lines, hypoxic responses were largely mediated by HIF-1α, while HIF-2α remained downregulated, possibly reflecting compensatory mechanisms previously described in these models [33].

In contrast, primary HCC cells expressed both HIF-1α and HIF-2α, a pattern previously associated with poor prognosis in HCC patients, highlighting fundamental differences in hypoxia signaling between model systems [34].

Metabolic analyses reveal that immortalized cell lines displayed relatively homogeneous adaptive behavior. Despite known differences in aggressiveness, HepG2 and Hep3B maintained coordinated glycolytic programs under hypoxia, characterized by increased glucose uptake and lactate secretion, albeit more pronounced in HepG2 than in Hep3B [35]. HepG2 exhibited a classical Warburg-like response under hypoxia [36], whereas Hep3B showed minimal metabolic plasticity, a feature previously reported under metabolic stress [37,38]. ATP levels were consistently higher under normoxia than hypoxia in both lines, consistent with the greater energetic efficiency of oxidative phosphorylation, while proliferative activity remained comparable between the two cell lines regardless of oxygen availability [39,40,41]. Collectively, these findings indicate that, within the immortalized HCC models analyzed, intrinsic aggressiveness does not markedly alter hypoxia-induced metabolic responses. In contrast, comparisons with patient-derived spheroids suggest that tumor aggressiveness may determine whether hypoxia induces active metabolic reprogramming or instead exposes a pre-existing metabolic configuration adapted to low-oxygen environments.

In contrast, patient-derived primary HCC cells preserved distinct malignancy-related metabolic phenotypes. The more aggressive HLC19 cells showed a glycolytic profile largely independent of oxygen availability, characterized by stable ATP levels, sustained proliferation and limited transcriptional remodeling [42,43,44,45,46]. This behavior is consistent with a state of intrinsic metabolic pre-adaptation to hypoxia as described in highly aggressive cancers [47]. Conversely, the less aggressive and more differentiated HLC21 cells displayed delayed and attenuated metabolic responses, with modest ATP increases only after prolonged hypoxic exposure, likely reflecting the compensatory engagement of residual oxidative metabolism [48]. Despite originating from advanced-stage tumors, HLC21 cells—being less aggressive and better differentiated—appeared metabolically more constrained, relying on balanced glucose utilization and producing less lactate [49,50].

Notably, hierarchical clustering based on the lactate-to-glucose uptake ratio grouped Hep3B with HLC19 and HepG2 with HLC21, independent of absolute metabolic activity or oxygen conditions. This observation suggests that metabolic strategy, rather than magnitude, aligns with aggressiveness: Hep3B and HLC19 preferentially channel glucose toward glycolysis, whereas HepG2 and HLC21 retain a more balanced, potentially oxidative metabolic profile. It should be noted that lactate-to-glucose uptake ratios occasionally exceeded the theoretical glycolytic stoichiometry of 2:1. This apparent supra-stoichiometric relationship likely reflects the contribution of alternative carbon sources, including glutamine metabolism or intracellular glucose reserves, as well as dynamic changes in extracellular metabolite pools during prolonged incubation. Similar observations have been reported in highly proliferative cancer models, where glycolytic and anaplerotic pathways operate simultaneously, supporting lactate production beyond direct glucose conversion [51]. Therefore, these ratios should be interpreted as indicators of metabolic strategy rather than strict stoichiometric measurements [51].

Gene expression analyses further reinforced the divergence among primary cells. HLC21 mounted a robust transcriptional response to hypoxia, with a marked induction of glycolytic genes, indicating active metabolic remodeling. In contrast, HLC19, already exhibiting a Warburg-like phenotype under normoxia, required minimal transcriptional reprogramming upon hypoxic exposure. Notably, GLUT1 and PKM2 were markedly upregulated in HLC21 cells, reflecting an ongoing metabolic shift towards glycolysis in response to oxygen deprivation. Together, these findings underscore how intrinsic aggressiveness in primary cells shapes both metabolic state and transcriptional plasticity.

Several limitations should be acknowledged. First, although the selected immortalized and primary HCC models represent well-characterized phenotypic contrasts in aggressiveness, the number of cell lines included is limited and does not capture the full biological heterogeneity of HCC. Therefore, our conclusions should be interpreted within the scope of the models analyzed and considered hypothesis-generating rather than universally generalizable.

Second, hypoxic exposure was limited to 48 h, allowing for the investigation of sustained hypoxic signaling and early-to-intermediate adaptive responses but not long-term evolutionary adaptation that may occur during extended hypoxic passaging. Future studies incorporating additional primary HCC models and prolonged hypoxic culture conditions will be important to further define the stability and conservation of the observed metabolic strategies.

Despite these limitations, the integration of functional metabolic measurements, transcriptional profiling, and phenotype-defined primary spheroids provides a controlled comparative approach for investigating aggressiveness-linked hypoxia adaptation.

These findings help us to interpret hypoxia-driven metabolic heterogeneity in HCC. Overall, our data support a model in which aggressive HCC cells may exist in a state of constitutive metabolic adaptation, characterized by high glycolytic activity and limited plasticity, whereas less aggressive tumors rely on inducible reprogramming to cope with hypoxic stress.

Importantly, patient-derived spheroids reveal biologically meaningful heterogeneity that is largely obscured in immortalized cell lines, highlighting their superior physiological relevance. These insights may have potential translational implications, as pre-adapted tumors may exhibit differential sensitivity to hypoxia- or metabolism-targeted therapies. Understanding aggressiveness-linked metabolic strategies may therefore inform the development of targeted metabolic therapies for HCC.

## 4. Materials and Methods

### 4.1. Immortalized and Primary Cell Lines

In this study we compared the effect of hypoxia in four HCC cell lines, two immortalized lines (HepG2 and Hep3B), and two patient-derived cells (HLC19 and HLC21). All cell lines were maintained at 37 °C in a humidified atmosphere containing 5% CO_2_. HepG2 and Hep3B cells, derived from hepatoblastoma and HCC respectively, are commonly described as less aggressive (HepG2) and more aggressive (Hep3B) models based on established differences in metabolic profiles, gene expression pattern and secretome composition [18,19]. HepG2 cells were purchased from the European Collection of Authenticated Cell Culture, while Hep3B were obtained from the American Type Culture Collection.

The primary cells HLC19 and HLC21 were kindly provided by Prof. G. Giannelli (National Institute of Gastroenterology IRCCS “Saverio de Bellis”, Research Hospital, Castellana Grotte). They were derived from surgical specimens of patients with histologically confirmed HCC, classified as Edmondson–Steiner grades IV (HLC19) and III (HLC21). All human samples were collected and manipulated following written informed consent from the patients and approval by the Institutional Review Board. This study was conducted in accordance with the 1975 Declaration of Helsinki and approved by the Institutional Ethics Committee of the National Institute of Gastroenterology IRCCS “Saverio de Bellis” (protocol code 1232/CE De Bellis—18 May 2023).

Both these cell lines have been previously characterized, and their biological differences are well documented [30]. Briefly, HLC21 displayed features such as sensitivity to anti-tumor treatments, whereas HLC19 cells exhibited an Edmondson–Steiner grades IV, fully mesenchymal phenotype, showing therapy resistance and enhanced stemness. In addition, we further analyzed these cells according to spheroid morphology, growth rate and migratory capacity (a shorter population doubling time and a greater migratory capacity in HLC19 than HLC21) as reported in the Supplementary Materials.

### 4.2. Spheroid Cultures of HCC Cells Under Hypoxic and Normoxic Conditions

3D spheroid cultures were generated from all cell lines using the same protocol. Briefly, cells were seeded at a density of 4 × 10^5^ cells per well in low-attachment plates in culture medium supplemented with 2% fetal bovine serum (FBS) and 1% penicillin/streptomycin. Cells were incubated for 3 days under standard conditions (37 °C, 5% CO_2_) to allow for self-assembly into spheroids. Immortalized cell lines were maintained in DMEM/F12 medium, while the primary HCC cells were maintained in Iscove’s Modified Dulbecco’s Medium (IMDM). After the formation of spheroids and 24 h of starvation, cultures were exposed to hypoxic conditions using a Modular Incubator Chamber (Embrient Inc., San Diego, CA, USA) set to 1% O_2_. Hypoxia was established by connecting the chamber to a gas mixture containing 1% O_2_, 10% CO_2_, and 89% N_2_. To remove residual oxygen present in the chamber and culture media, the chamber was flushed at a flow rate of 20 L/min for 7 min and then placed in a standard incubator. Oxygen levels in the culture medium were monitored using an oxygen sensor chip positioned at the bottom of each well and connected to a fiber-optic probe and data acquisition system (PreSens Precision Sensing GmbH, Regensburg, Germany) at baseline and after 2, 4, 24 and 48 h of culturing. Normoxic conditions of culture (21% O_2_) were used as a comparison.

### 4.3. Quantification of Glucose Consumption and Lactate Production

To assess the metabolic activity of HCC spheroids under hypoxic and normoxic conditions, glucose consumption and lactate production were measured. After spheroid formation, cultures were incubated in starvation medium (same culture medium without serum) for 24 h, to normalize the activity of all cells. Spheroids were then transferred to adhesion plates in DMEM/F12 medium containing 5 mM glucose, 2 mM glutamine and 10% dialyzed FBS for 2, 4, 6, 24 and 48 h under hypoxic and normoxic conditions. At each time point, the culture supernatant was collected, diluted in phosphate-buffered saline (PBS) and stored at −20 °C until analysis. In parallel, cells were inactivated and neutralized using specific acid buffers and stored for the viability assay below.

Extracellular glucose was measured with Glucose-Glo™ Assay (Promega, Milan, Italy) after the dilution of samples in PBS (1:200). Glucose uptake was calculated as the difference between glucose concentration in fresh cell free-medium (baseline) and that measured in supernatants. Lactate secretion was quantified in the supernatant using the Lactate-Glo™ Assay (Promega, Milan, Italy) after dilution in PBS (1:100), subtracting baseline lactate levels present in the medium.

All assays were performed according to the manufacturer’s instructions. Standard curves for glucose and lactate were generated and loaded onto opaque plates for the quantification of these metabolites, expressed in μM. Luminescence was measured using GloMax^®^ Discover Microplate Reader (Promega, Milan, Italy) after shaking for 5 min and incubation for 1 h at RT. All experiments were performed in at least three independent biological replicates with technical duplicates or triplicates. Based on the metabolic measurements, the lactate secretion-to-glucose uptake ratio was calculated, and these ratios were clustered using Python-based analysis (version 3.10) implemented in Google Colab [52].

### 4.4. ATP Production of HCC Spheroids Under Hypoxic and Normoxic Conditions

3D spheroids from all cell types were also evaluated for ATP production under the same hypoxic and normoxic conditions used for the metabolic assays. At defined time points (2, 4, 6, 24 and 48 h of culture), spheroid lysates obtained during the metabolic assay (as previously described) were collected, stored at −20 °C and subsequently used to assess cell viability and energy status.

This approach enabled the direct evaluation of the viability of the cells analyzed in the metabolic assays and allowed for the correlation of glucose uptake and lactate secretion with cellular ATP production. Samples were transferred to white opaque plates, and ATP-dependent luminescence was measured by adding the CellTiter-Glo^®^ 3D Cell Viability Assay buffer (Promega, Milan, Italy). Luminescence was measured using GloMax^®^ Discover Microplate Reader (Promega, Milan, Italy) after shaking for 5 min and incubation for 25 min at RT. Data are reported in graphs as Relative Light Units (RLU).

### 4.5. Cell Proliferation of HCC Spheroids Assessed by Immunofluorescence Assay

Immortalized and primary cells were seeded at a density of 5 × 10^4^ cells per well in low-attachment plates in complete medium supplemented with 2% FBS to generate spheroids. After formation, starved 3D spheroids were transferred to black adhesion plates containing complete medium supplemented with 10% FBS and incubated under hypoxic or normoxic conditions, as described above. After 2 and 48 h of culture, spheroids were fixed in 4% formaldehyde to assess cell proliferation by the immunofluorescent detection of Ki67. Following blocking with Image IT Fix (Invitrogen, Waltham, MA, USA), spheroids were incubated with anti-Ki67 primary antibody (D3B5, Cell Signaling Technology, Danvers, MA, USA), followed by fluorochrome-conjugated secondary antibodies (Alexa Fluor 488, Life Technologies, Carlsbad, CA, USA). Nuclei were counterstained with DAPI (Thermo Fisher Scientific).

Z-stack images of spheroids were acquired using a Leica MICA microscope in confocal mode. Quantitative image analysis was performed using FIJI software (ImageJ 2.16.0/1.54p). Briefly, fluorescence images were converted to grayscale and subjected to automatic thresholding to segment DAPI-stained nuclei. Nuclei were counted using the “Analyze Particles” function. Subsequently, Ki67 signal intensity was measured within the same nuclear regions using the “Multi-Measure” function. Data are expressed as the percentage of Ki67-positive cells per spheroid. Each experiment was performed at least three independent times, and three or more spheroids per well were analyzed for quantification.

### 4.6. Gene Expression Analysis by Digital PCR

Spheroids were generated from all cell lines by seeding 4 × 10^5^ cells per well. After formation and starvation, 3D spheroids were transferred to adhesion plates in complete medium with 10% of FBS and incubated under hypoxic or normoxic conditions, as described above. Total RNA was extracted from 3D spheroids with Nucleozol (Mechery-Nagel GmbH & Co. KG, Dueren, Germany) and purified with NucleoSpin columns (Mechery-Nagel GmbH & Co. KG). RNA concentration was quantified, and complementary DNA (cDNA) was synthesized according to the manufacturer’s instructions.

Digital PCR (dPCR) reactions were prepared using ddPCR Supermix (Bio-Rad, Segrate, Milan, Italy), gene-specific primers (Thermo Fisher Scientific, Waltham, MA, USA) and cDNA samples. After amplification, the droplets for each well were read automatically with QX200 Droplet Reader (Bio-Rad). All procedures were performed according to the manufacturer’s instructions. Candidate reference genes were evaluated for expression stability in all cell lines using RefFinder [53], and the most stable gene within each cellular group was selected for normalization (as reported in Supplementary Materials and Figure S3).

Absolute transcript levels (copies/µL) were normalized to reference genes (YWHAZ for immortalized cell lines and POLR2A for primary cell lines), log_2_-transformed and plotted following statistical analysis. The target genes analyzed included HIF-1α, HIF-2α, CA9, EGR1, VEGFA, GLUT1, PDK1, LDHA, and PKM2 (Thermo Fisher Scientific, Waltham, MA, USA). All experiments were performed in at least three independent replicates, with three technical replicates for each.

### 4.7. Statistical Analysis

The results were expressed as means ± SEM (standard error of the mean). Each experiment was repeated at least three times. Differences between groups were tested for statistical significance with a Two-way ANOVA followed by appropriate post hoc tests (Tukey’s multiple comparison test) for multiple comparisons, using GraphPad software (Prism 9, San Diego, CA, USA). Differences were considered significant at a *p* value < 0.05. For the metabolic data, we performed Pearson correlation to assess the relationship between glucose uptake and lactate secretion under normoxia and hypoxia. Positive correlation was considered significant at a *p* value < 0.05.

## Figures and Tables

**Table 1:** **Pearson correlation coefficients (r) between glucose uptake and lactate secretion in HCC cell lines under normoxia and hypoxia.** Correlation analyses were performed for two primary (HLC19, HLC21) and two immortalized (HepG2, Hep3B) HCC cell lines. A strong positive correlation was observed in primary cells under both normoxic and hypoxic conditions, whereas among immortalized lines, a significant correlation was detected only for HepG2 under hypoxia. Significant correlations are shown in bold. Statistical significance: *p* < 0.05; *p* < 0.01; *p* < 0.001.

Cell Line	Condition	Pearson R	*p*-Value
HepG2	Normoxia	0.203	0.390
Hep3B	Normoxia	−0.492	0.053
**HLC19**	**Normoxia**	**0.884**	**<0.001**
**HLC21**	**Normoxia**	**0.662**	**0.007**
**HepG2**	**Hypoxia**	0.717	**<0.001**
Hep3B	Hypoxia	0.014	0.953
**HLC19**	**Hypoxia**	**0.789**	**<0.001**
**HLC21**	**Hypoxia**	**0.933**	**<0.001**

## Data Availability

All data and materials used are available within the published manuscript and in its Supplementary Information Files.

## Supplementary Materials

The following supporting information can be downloaded at https://www.mdpi.com/article/10.3390/ijms27073069/s1.

## Abbreviations

The following abbreviations are used in this manuscript:

ATP	Adenosine triphosphate
CA9	Carbonic anhydrase IX
DAPI	4′, 6–diamidino-2-phenylindole
DMEM	Dulbecco’s modified eagle medium
dPCR	Digital polymerase chain reaction
EGR-1	Early growth response 1
EMT	Epithelial–mesenchymal transition
FBS	Fetal bovine serum
GLUT1	Glucose transporter type 1
HCC	Hepatocellular carcinoma
HIF-1α	Hypoxia-inducible factor 1-alpha
HIF-2α	Hypoxia-inducible factor 2-alpha
IMDM	Iscove’s modified Dulbecco’s medium
LDHA	Lactate dehydrogenase A
PBS	Phosphate-buffered saline
PDK1	3-phosphoinositide-dependent kinase-1
PKM2	Pyruvate kinase M2
PDT	Population doubling time
POLR2A	RNA polymerase II subunit A
TACE	Transarterial chemoembolization
VEGFA	Vascular endothelial growth factor A
YWHAZ	Tyrosine 3-monooxygenase activation protein zeta
