# Supplementary material for: Intrinsic Tumor Aggressiveness Dictates Hypoxia-Driven Metabolic Programs in Hepatocellular Carcinoma
Source: Int J Mol Sci. 2026 Mar 27;27(7):3069. doi: 10.3390/ijms27073069 (PMC13073595; doi:10.3390/ijms27073069)
Supplement: Supplementary file 1 [file ijms-27-03069-s001.zip › ijms-4177250-supplementary.pdf]

International Journal of
Molecular Sciences

MDPI

S1. Supplementary Methods

S1.1. Morphology and proliferation rate analysis of primary cell lines.

HLC19 and HLC21 cells were maintained in two-dimensional (2D) culture under standard conditions (5% of CO_2, 37° C) at density of 2x10^6 cells and were observed at 24 h and 48 h post-seeding, to evaluate the differences between cells in morphology and growth rate. At each time point, at least three images per cell line were acquired using a Leica DM camera and detached cells were counted using a Boyden chamber to calculate growth rate, expressed as population doubling time (PDT). The latter was calculated using the following formula:

$$PDT = \frac{\log_{(10)} 2 \cdot \Delta T}{\log_{(10)} N_T - \log_{(10)} N_{T_0}}$$

T was the time expressed in days, N(T) was the number of cells after 24 h or 48 h post-seeding and N(T0) was the number of cells at baseline [Pisciotta, A.; Riccio, M.; Carnevale, G.; Beretti, F.; Gibellini, L.; Maraldi, T.; Cavallini, G.M.; Ferrari, A.; Bruzzesi, G.; De Pol, A. Human Serum Promotes Osteogenic Differentiation of Human Dental Pulp Stem Cells in Vitro and in Vivo. PLoS ONE 2012, 7, e50542. https://doi.org/10.1371/journal.pone.0050542.; Greenwood, S.K.; Hill, R.B.; Sun, J.T.; Armstrong, M.J.; Johnson, T.E.; Gara, J.P.; Galloway, S.M. Population Doubling: A Simple and More Accurate Estimation of Cell Growth Suppression in the in Vitro Assay for Chromosomal Aberrations That Reduces Irrelevant Positive Results. Environ. Mol. Mutagen. 2004, 43, 36–44. https://doi.org/10.1002/em.10207]. Qualitative and quantitative results were reported in Figure S1.

S1.2. Migration assay.

Fifty-thousand cells per well were seeded in low-attachment plates for three days in order to form spheroids from patient-derived cells. The obtained spheroids were transferred into attachment plates after 24 h of starvation, at standard culture conditions (5% of CO_2 and 37° C). In the migration assay, to give the spheroids enough time to adhere, we started the observation 3 h after seeding and, as additional time points, after 24 and 48 h. At each time point, images were acquired with the MICA microscope (Leica, Wetzlar, Germany) in widefield mode. We measured the spheroid area using the Fiji ImageJ tool as an indicator of migration capability, as our models did not clearly distinguish between the spheroid core and the migration area. Results were represented as bar graphs following statistical analysis.

S2. Supplementary Results

S2.1. HLC19 cells exhibit higher proliferative capacity and mesenchymal features

The morphology analysis indicated that HLC19 cells exhibited an elongated phenotype with extensions or protrusions, which may indicate motility (Figure S1 A, C); in addition, HLC21 appeared less compact than hepatocytes and more elongated and spindle-shaped (Figure S1 B, D). Neither cell line formed compact clusters indicating the transition to a potential mesenchymal phenotype. HLC19 cells showed significantly shorter PDT compared with HLC21 cells (Figure S1 E). This difference was consistent across four independent experiments ($p = 0.015$), indicating a higher proliferative capacity of the HLC19

line under the tested conditions. The combined phenotypic analysis suggests a more differentiated and less aggressive phenotype of HLC21 compared with HLC19 which likely exhibits a partial EMT phenotype, associated with metastatic potential of the cells.

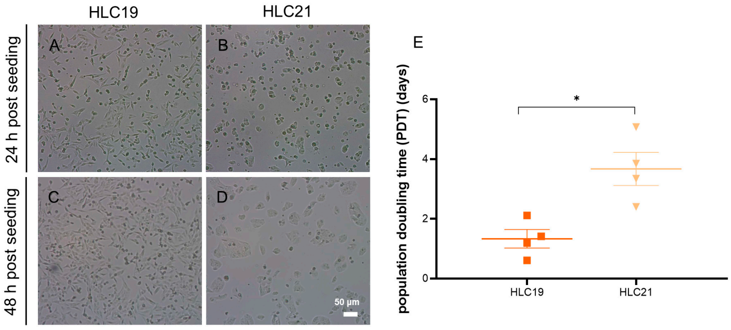

Figure S1. Qualitative and quantitative analysis of HLC19-HLC21's morphology and proliferation. Representative images of HLC19 at 24 h (A) and 48 h (C) of culture in 2D after seeding and of HLC21 at the same time points (at 24 h (B) and 48 h (D)). Data of population doubling time (PDT) was reported as means ± SEM (E). Statistical comparison: * $p < 0.05$.

S2.2. HLC19 cells have greater migratory capacity than HLC21

In the migration assay of HLC19 and HLC21 cells, we observed that the two cell lines, which appear similar after 3 h, have two different phenotypes after 24 and 48 h post-seeding. HLC19 spheroids lost their spherical morphology already after 24 h with a significant increase in spheroid size versus 3 h (Figure S2 A, B). Moreover, the comparison between HLC19 and HLC21 revealed that HLC19 had a significant increase at 24 and 48 h compared with HLC21. These findings support the use of HLC19 and HLC21 as in vitro models of hepatocyte-derived cells with distinct biological properties and differential cellular aggressiveness.

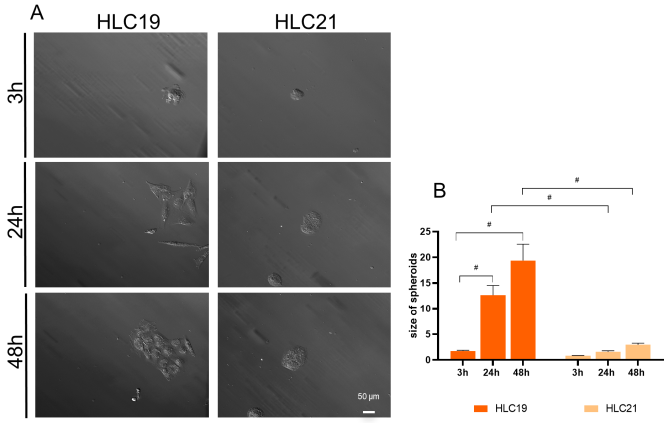

Figure S2. Migration assay of HLC19 and HLC21 spheroids under standard culture conditions. (A) Representative images of HLC19 and HLC21 spheroids after 3, 24 and 48 h of adhesion in a plate. (B) Quantification data of spheroid's size as indicator of migration capability. Statistical comparison: # $p < 0.0001$.

S2.3. Preliminary stability analysis of reference genes

https://doi.org/10.3390/ijms27073069

To ensure accurate normalization of gene expression data, several candidate reference genes (YWHAZ, HMBS, POLR2A, and TBP) were initially evaluated for expression stability across the four HCC cell lines used in this study, including two immortalized (HepG2 and Hep3B) and two patient-derived primary models (HLC19 and HLC21). Stability analysis was performed using the RefFinder algorithm [55], which integrates multiple computational approaches to rank candidate reference genes according to their expression stability. This analysis identified YWHAZ as the most stable reference gene in immortalized cell lines and POLR2A as the most stable in primary HCC cells (Supplementary Figure 3). Furthermore, we observed no significant variations in their expression across all experimental culture conditions, including normoxia and hypoxia at different time points in each group of cells. Accordingly, YWHAZ and POLR2A were selected for normalization of gene expression data within their respective cellular groups.

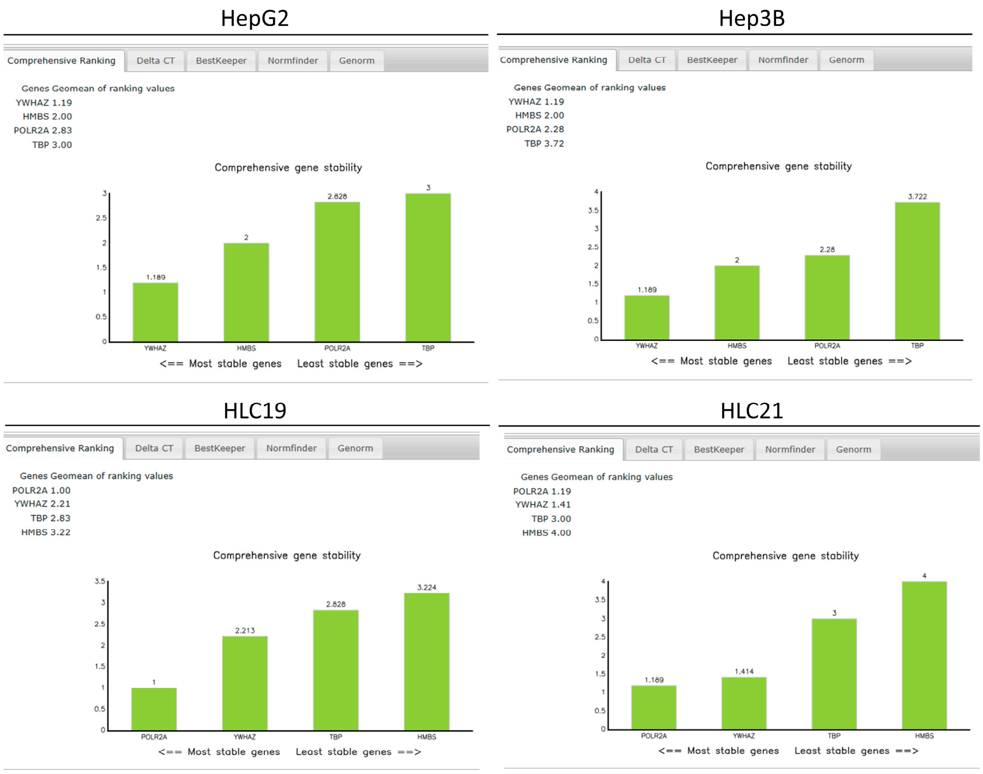

Figure S3. Reference genes expression stability. Comprehensive ranking of gene stability in HepG2, Hep3B, HLC19 and HLC21 for YWHAZ, HMBS, POLR2A, and TBP genes. YWHAZ is the most stable gene in HepG2 and Hep3B cells, while POLR2A in HLC19 and HLC21 cells.

S2.4. Summary of metabolic, viability and proliferative assay data

This study investigated the impact of hypoxia on the energetic metabolism and cellular activation of HCC cells, focusing on differences related to tumor aggressiveness. Metabolic, viability and proliferation data were directly compared between more and less aggressive cell lines within immortalized and primary models. Under hypoxic conditions, glucose uptake showed a similar temporal trend in all cell lines, with no significant differences, although a late increase was observed in immortalized cells (Figure S4A). A similar pattern was observed under normoxia with significant increase only in primary cells, according to aggressiveness (Figure S5A). In contrast, lactate secretion under hypoxia was significantly higher in HLC19 cells than HLC21 at the final time point under both conditions (Figure S4B, Figure S5B) whereas no differences were observed between immortalized cells. More pronounced differences emerged in viability and proliferation. Under normoxia but also under hypoxia, the HLC19 spheroids displayed consistently

higher viability over time and a greater proportion of proliferative cells at 48 h compared with HLC21 (Figure S4C-D; Figure S5C-D). These differences were not so clear in the immortalized group. Collectively, these results indicate that HLC19 cells are intrinsically better adapted to hypoxic stress, displaying metabolic and proliferative programs that sustain survival and growth under low oxygen conditions, while HLC21 cells show a weaker adaptative response.

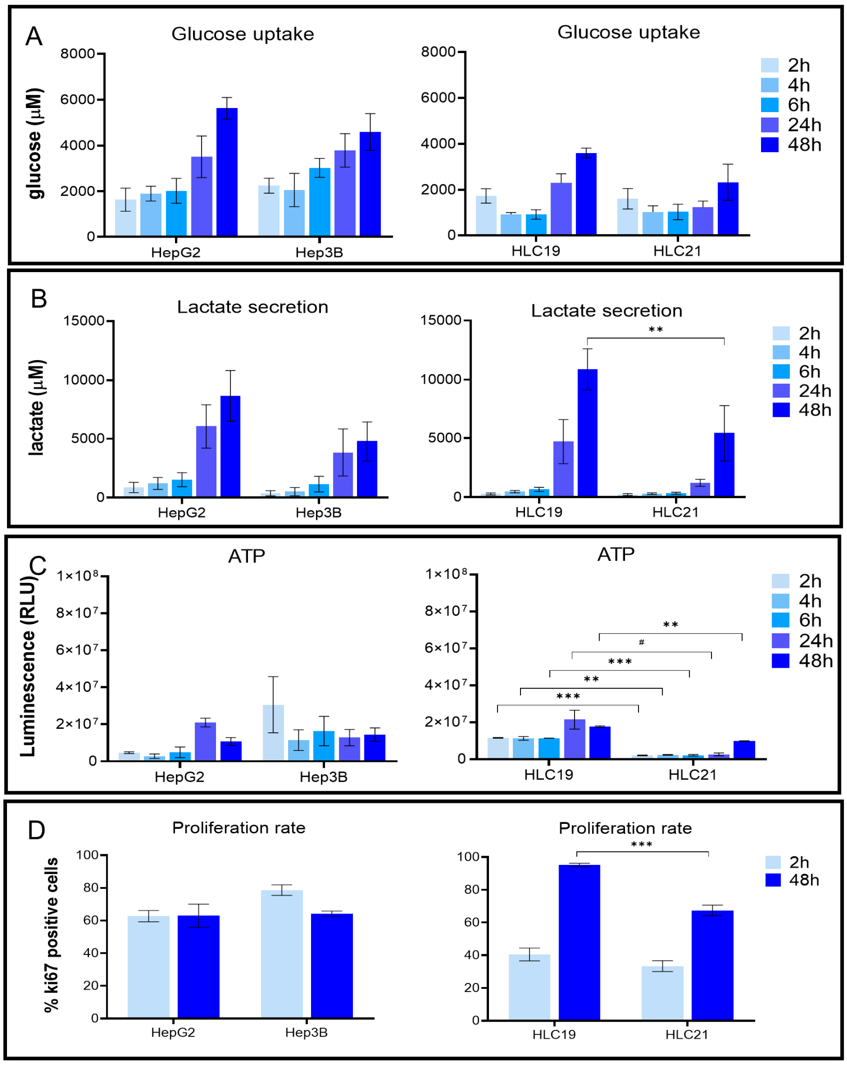

Figure S4. Comparative summary of data obtained from hypoxic conditions of culture of all cell lines. (A) Glucose uptake measured over time in immortalized (HepG2, Hep3B) and primary (HLC19, HLC21) HCC cells cultured under hypoxia. (B) Lactate secretion levels in the same cell models, reflecting glycolytic activity during hypoxic exposure; (C) Intracellular ATP production in the same cell models assessed as a measure of energetic output in response to low-oxygen conditions; (D). Proliferative activity of all spheroids evaluated by quantification of Ki67-positive cells, showed differential growth dynamics between more and less aggressive HCC cell lines under hypoxia. Statistical significance: ** $p < 0.01$; *** $p < 0.001$; # $p < 0.0001$.

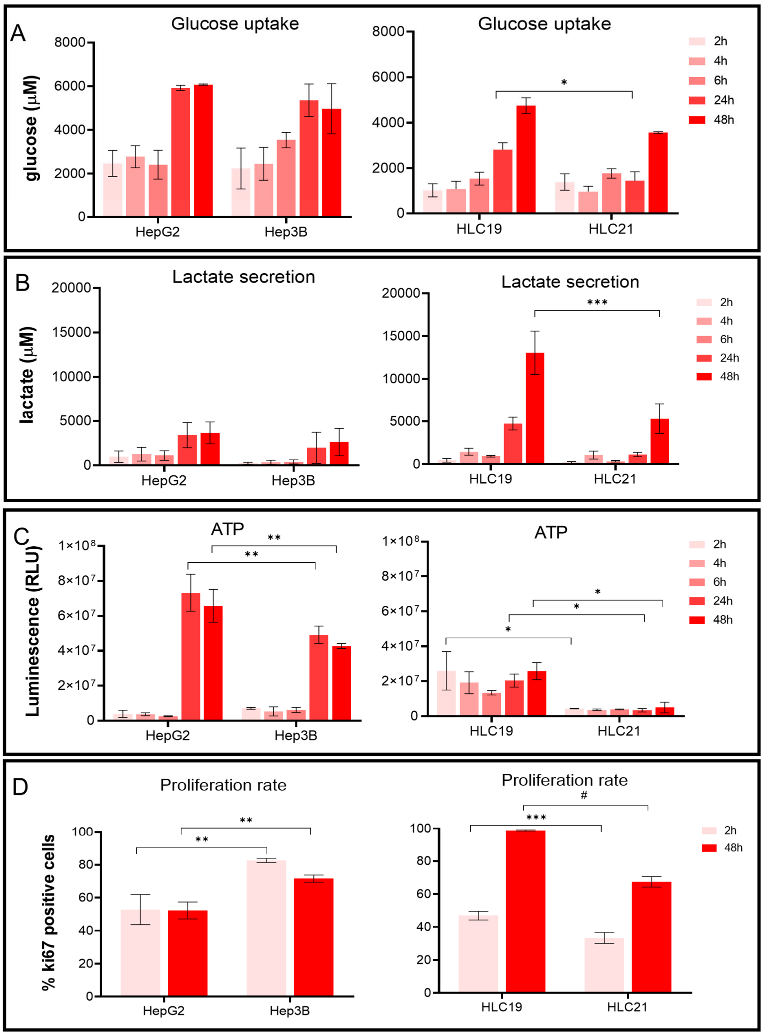

Figure S5. Comparative summary of data obtained from normoxic conditions of culture. (A) Glucose uptake measured over time in immortalized (HepG2, Hep3B) and primary (HLC19, HLC21) HCC cells cultured under normoxia. (B) Lactate secretion levels in the same cell models, reflecting glycolytic activity during normoxic exposure, more pronounced in HLC19. (C). Intracellular ATP production assessed to evaluate energetic output in response to standard-oxygen conditions; (D). Proliferation rate analysis, by quantification of Ki67-positive cells, showed differential growth dynamics between more and less aggressive HCC cells under normoxia. Statistical comparison: * $p < 0.05$, ** $p < 0.01$; *** $p < 0.001$; # $p < 0.0001$.
