# Supplementary figures and images for: Intrinsic Tumor Aggressiveness Dictates Hypoxia-Driven Metabolic Programs in Hepatocellular Carcinoma
Source: Int J Mol Sci. 2026 Mar 27;27(7):3069. doi: 10.3390/ijms27073069 (PMC13073595; doi:10.3390/ijms27073069)

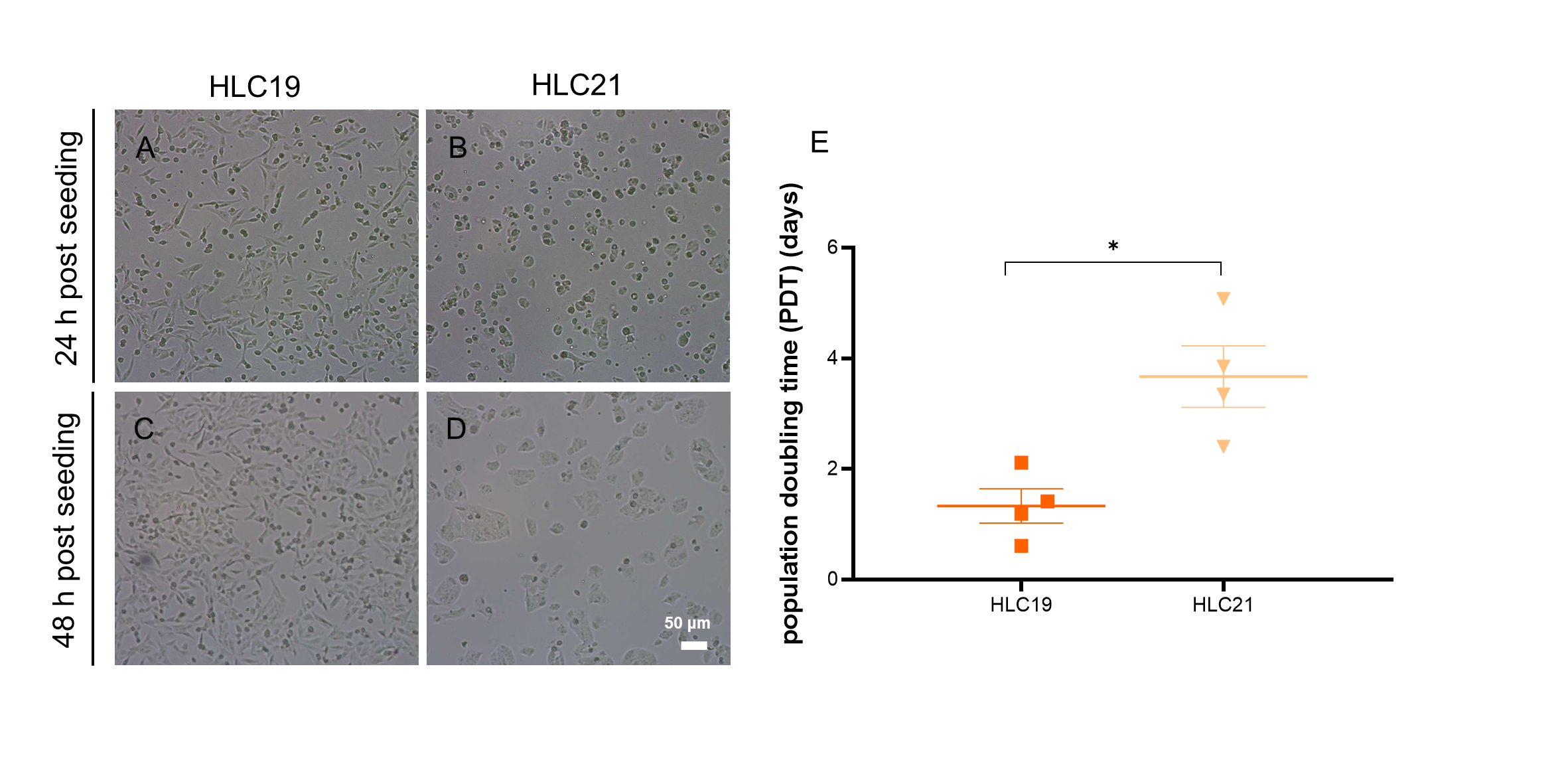

Supplement: Supplementary file 1 [file ijms-27-03069-s001.zip › FigureS1.tif]

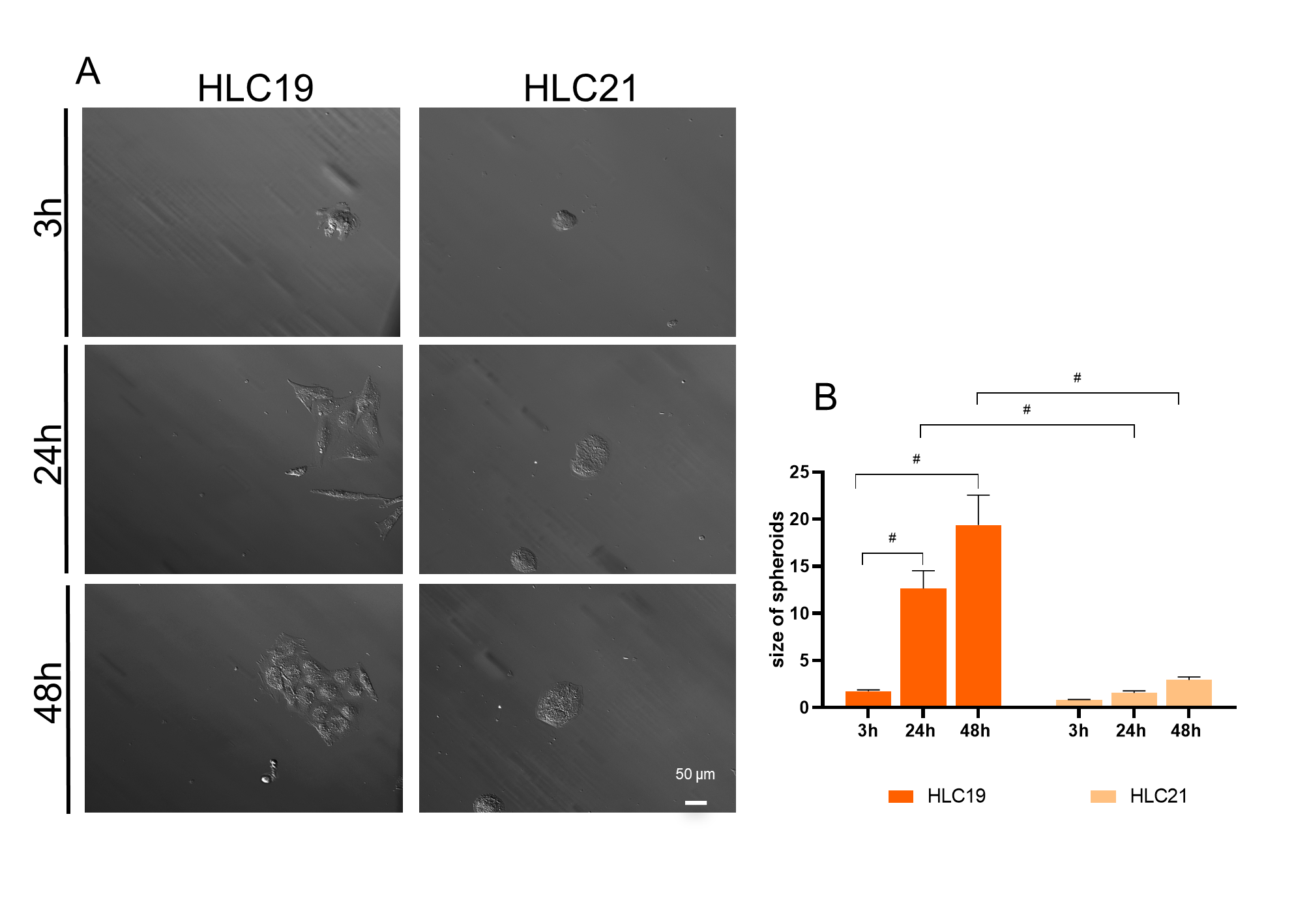

Supplement: Supplementary file 1 [file ijms-27-03069-s001.zip › FigureS2.tif]

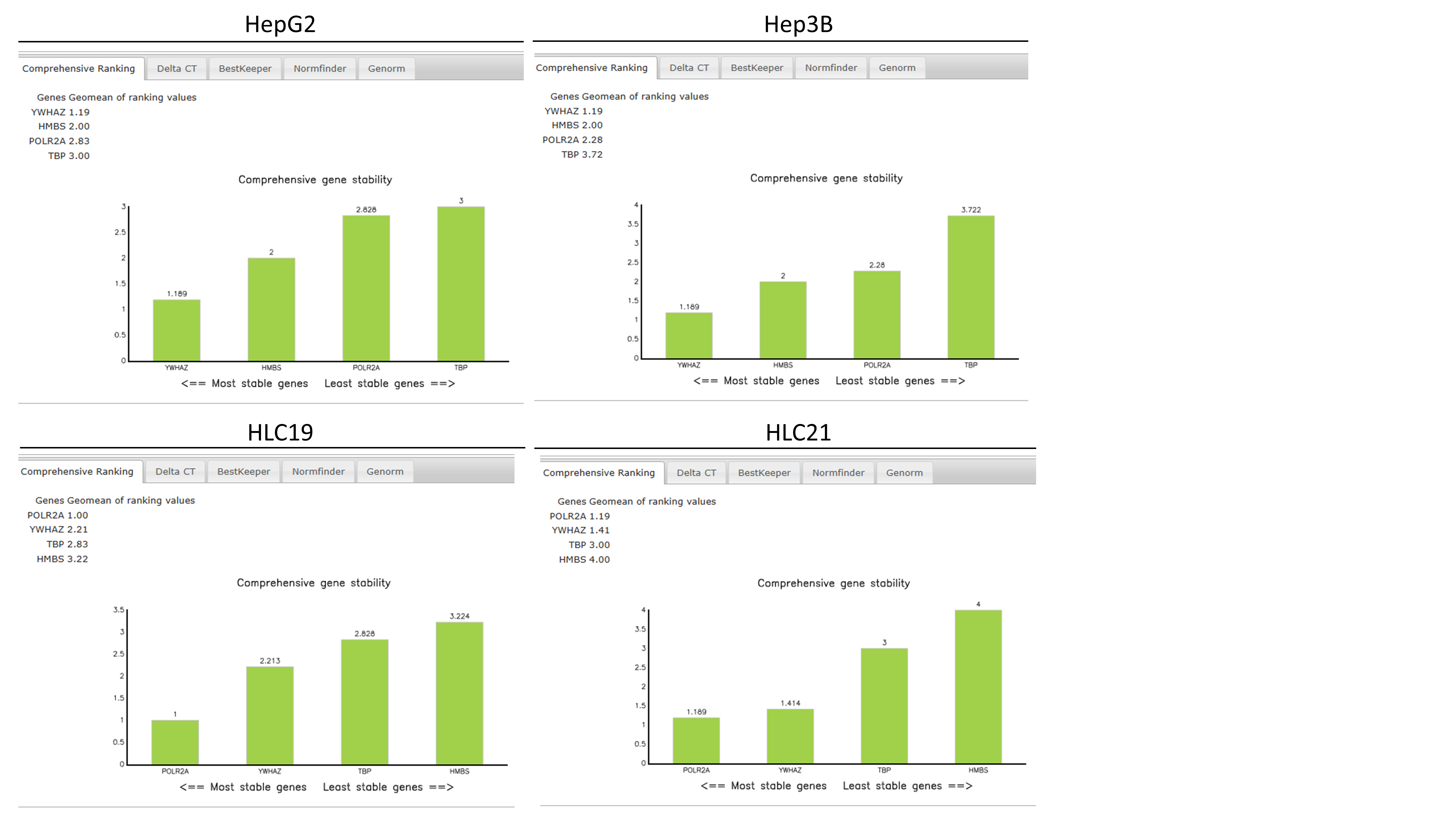

Supplement: Supplementary file 1 [file ijms-27-03069-s001.zip › FigureS3.tif]

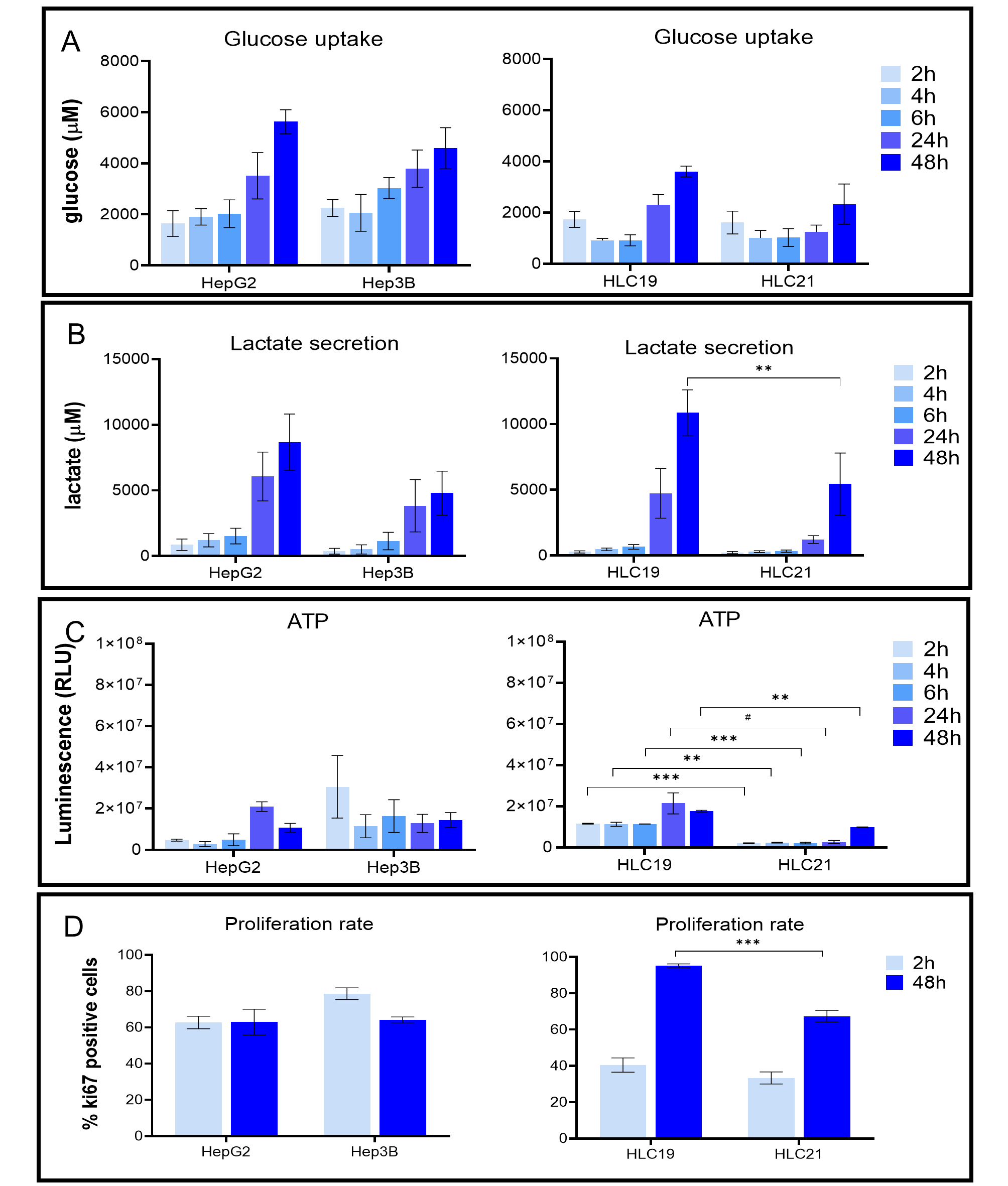

Supplement: Supplementary file 1 [file ijms-27-03069-s001.zip › FigureS4.tif]

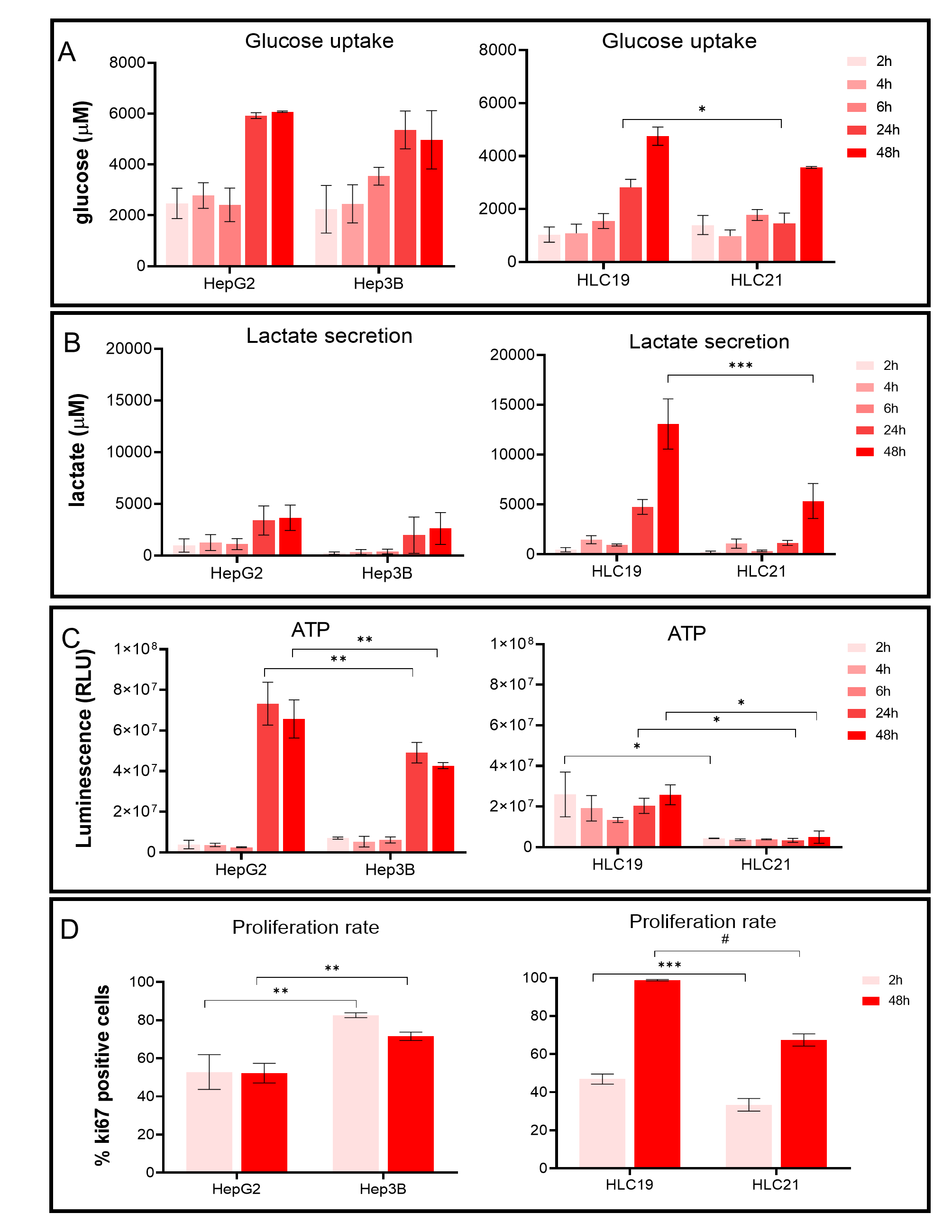

Supplement: Supplementary file 1 [file ijms-27-03069-s001.zip › FigureS5.tif]
